# Analysis of generic coupling between EEG activity and P_ET_CO_2_ in free breathing and breath-hold tasks using Maximal Information Coefficient (MIC)

**DOI:** 10.1038/s41598-018-22573-6

**Published:** 2018-03-14

**Authors:** Maria Sole Morelli, Alberto Greco, Gaetano Valenza, Alberto Giannoni, Michele Emdin, Enzo Pasquale Scilingo, Nicola Vanello

**Affiliations:** 1 Department of Life science, Scuola Superiore Sant’Anna, Pisa, 56127 Italy; 2 Research Center “E. Piaggio”, University of Pisa, Pisa, 56122 Italy; 3 Dipartimento di Ingegneria dell’Informazione, University of Pisa, 56124 Pisa, Italy; 4 Fondazione Toscana Gabriele Monasterio, National Research Council, 56124 Pisa, Italy

## Abstract

Brain activations related to the control of breathing are not completely known. The respiratory system is a non-linear system. However, the relationship between neural and respiratory dynamics is usually estimated through linear correlation measures, completely neglecting possible underlying nonlinear interactions. This study evaluate the linear and nonlinear coupling between electroencephalographic (EEG) signal and variations in carbon dioxide (CO_2_) signal related to different breathing task. During a free breathing and a voluntary breath hold tasks, the coupling between EEG power in nine different brain regions in delta (1–3 Hz) and alpha (8–13 Hz) bands and end-tidal CO_2_ (P_ET_ CO_2_) was evaluated. Specifically, the generic associations (i.e. linear and nonlinear correlations) and a “pure” nonlinear correlations were evaluated using the maximum information coefficient (MIC) and MIC-*ρ*^2^ between the two signals, respectively (where *ρ*^2^ represents the Pearson’s correlation coefficient). Our results show that in delta band, MIC indexes discriminate the two tasks in several regions, while in alpha band the same behaviour is observed for MIC-*ρ*^2^, suggesting a generic coupling between delta EEG power and P_ET_CO_2_ and a pure nonlinear interaction between alpha EEG power and P_ET_CO_2_. Moreover, higher indexes values were found for breath hold task respect to free breathing.

## Introduction

The mechanism underlying breathing control is not totally understood at the moment. Several mathematical models have been developed to describe the control of breathing, following the hypothesis that the respiratory system could be considered as a closed loop, with a specific transit time^1^. In these models, the control of ventilation is described considering the relationship between the changes in carbon dioxide (CO_2_) and oxygen (O_2_) and ventilation. Some models describe the dynamics between CO_2_ and ventilation using a linear interaction^2–4^. However, this assumption does not consider that the respiratory system is a non-linear system^5^, especially moving away from the steady state, as in oscillatory phenomena, such as periodic breathing or central/obstructive apneas. For this reason, some models hypothesized a non-linear relationship between the changes in CO_2_ and ventilation^6–8^.

A fundamental role in the control of breathing is played by the central nervous system (CNS). It is known that the breathing control originates in the brainstem^9^. In these brain areas, the central chemoreceptors are sensitive to CO_2_ variation. Specifically, when an increase in arterial CO_2_ level occurs (hypercania) a subsequent increase in ventilation is observed^1^ to maintain homoeostatic level of gases in blood^10^, both in health^11^ and disease^12^. Not only the brainstem, but also cortical and subcortical areas are involved in breathing processes^13–17^. Generally, hypercapnia also causes vasodilation, and it is responsible of a certain number of vascular changes in the brain, such as variation in cerebral blood flow and cerebral blood volume^18^, usually following linear dynamics. On the other hand, considering the neural activity related to hypercapnia, different effects have been observed. Neuronal oscillatory power is strongly linked to arterial CO_2_ increase^19^ and both linear and non linear relationship have been hypothesized^9^. Another difference between the vascular and neural pattern of CO_2_ response is that the former is mainly widespread while the latter may be regionally heterogeneous.

The effect of hypercapnia on brain activity has been explored by gas administration using electroencephalography (EEG)^20,21^. Hypercapnia reduces spontaneous neuronal oscillatory power in anaesthetized primate^22^ and rats with intracortical electrodes^23^ and, if prolonged (8 weeks), causes hypnotic effects without changing the morphological aspect of the brain in rabbits^24^. In humans, it is responsible for increasing the *δ* band (1–4 Hz) EEG power as well as for decreasing the power in the *α* (8–13 Hz) band^25,26^, suggesting that during hypercapnic inhalation, brain activity resembles low arousal state. Furthermore, it has been observed a reduction in EEG power not only in *α*- but also in *β*-, and *γ*-frequency bands^22,27^.

Voluntary breath hold can be considered an alternative to gas induced hypercapnia to evaluate the relationship between CO_2_ and brain activity variations. Indeed, voluntary breath hold is easy to perform and has the advantage to reach arterial hypercapnia without requiring a specific device for the CO_2_ administration^28^. This kind of task allows to observe the effect of a progressive increase in arterial CO_2_, differently from gas administration where a step increase (square wave) to supraphysiological CO_2_ ranges is often achieved. Moreover, it resembles some features of the respiratory dynamics observed in obstructive sleep apnea (OSA) and central sleep apnea (CSA), where a sinusoidal increase of about 1 kPa (that correspond to about 7.5 mmHg) is usually recorded^29,30^ during an average apnea length around 30 seconds^31–33^. Obviously, the respiratory cycle timing is fundamental to stress the chemoreflex system in the right range of perturbation. While, past studies used too long (80–225 seconds) or too short (10 seconds) voluntary breath hold intervals^34,35^, a more recent study by our group explored the cross correlation between EEG global field power (GFP) in *δ* band and end-tidal CO_2_ (P_ET_CO_2_) during 30 seconds of breath hold finding that the variation of P_ET_CO_2_ precedes the variations of GFP^36^. However, in^36^ only a linear relationship between the two signal was considered, studying only the Pearson coefficient (*ρ*) at different time shifts and completely excluding non-linear interactions. Further, only the global EEG power was analysed without considering potential different responses in regional cortical areas.

To capture both linear and non-linear coupling between two variables it is possible to compute the maximal information coefficient (MIC)^37^. MIC was proved to be a valid index to construct the brain functional network^38^. It was used to highlight the non-linearity of neurovascular coupling between EEG and BOLD signals in a simultaneous EEG-fMRI study^39^. Moreover, MIC was used to observe the brain-heart interaction in volunteers during viewing of pictures from the International Affective Picture System (IAPS)^40^.

Here, we want to study the complex relationship between P_ET_CO_2_ and EEG power variations, using MIC analysis. Indeed, MIC includes both linear and non-linear relationship and for this reason also MIC-*ρ*^2^ has been considered as a measure of “pure” non-linear interactions. We evaluated the effects of breath hold task locally, on nine different brain regions obtained dividing the scalp in 3 rostrocaudal sections (anterior, central, and posterior) and 3 sagittaly sections (left, middle, and right). Understanding both linear and non-linear mechanisms underlying the neuronal response to hypercapnia at regional level could be useful to disentangle the vascular and neuronal response to CO_2_ variations.

## Results

**Figure 1:**
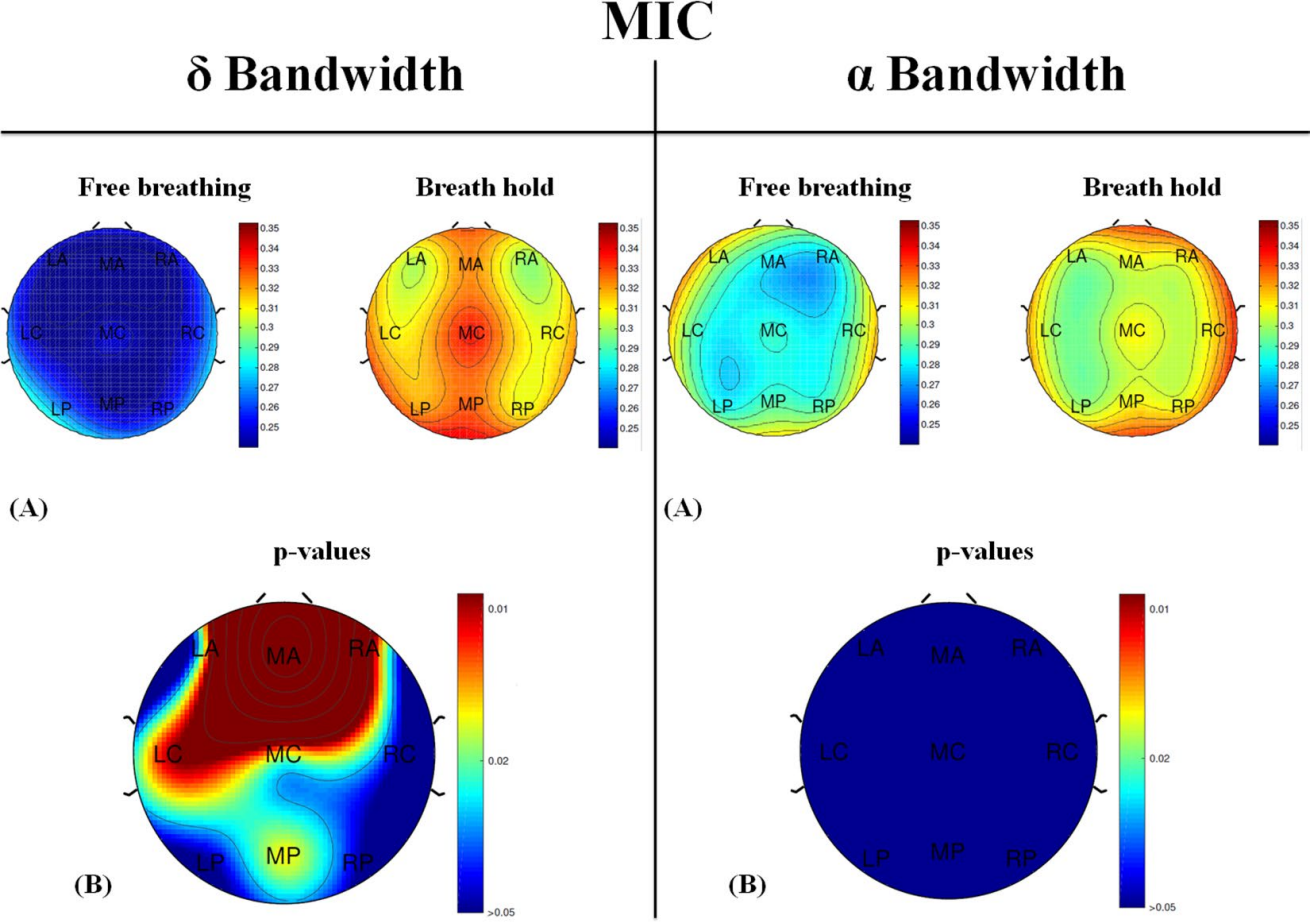
(**A**) Topographic Map of MIC values computed in 9 different areas (3 rostrocaudal sections -anterior, central, and posterior- and 3 sagittaly sections -left, middle, and right) in *δ* (left) and *α* (right) bands during the two tasks: free breathing (FB) and breath hold (BH). (**B**) Topographic Maps of p-values resulting from the Wilcoxon signed rank test judged by a significance level of 0.05 between FB- and BH-MIC computed in *δ* (left) and *α* (right) bands.

**Figure 2:**
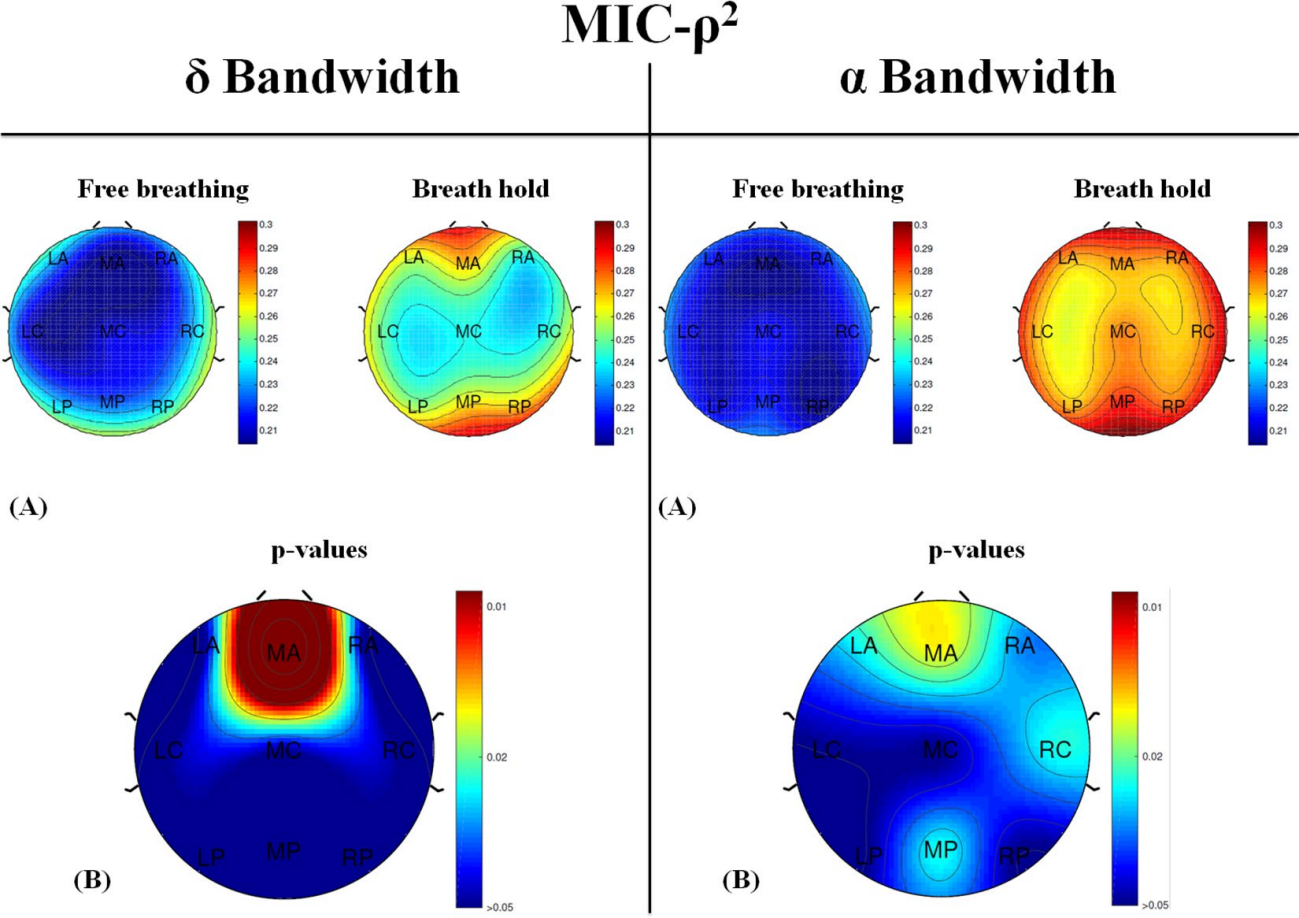
(**A**) Topographic Map of MIC-*ρ*^2^ values computed in 9 different areas (3 rostrocaudal sections -anterior, central, and posterior- and 3 sagittaly sections -left, middle, and right) in *δ* (left) and *α* (right) bands during the two tasks: free breathing (FB) and breath hold (BH). (**B**) Topographic Maps of p-values resulting from the Wilcoxon signed rank test judged by a significance level of 0.05 between FB- and BH- MIC-*ρ*^2^ computed in *δ* (left) and *α* (right) bands.

Observing SpO_2_ values, both FB and BH tasks did not generate variation in oxygen saturation level in healthy subjects. However, observing the P_ET_CO_2_ signal, specifically the variation in P_ET_CO_2_, in FB a mean value of 5.1 ± 2.1 mmHg was estimated, respect to BH task where a mean value 11.0 ± 2.7 mmHg was found. Since the segments with not reducible artefacts (bad blocks) were excluded from the analysis, the proportion of valid EEG signal across subjects was comprised between 50% and 95% of the total signal (i.e., between 9000 and 17100 samples). Surrogate analysis tests confirmed that the estimated MIC values are significant. Specifically, the lowest significance threshold was found to be equal to 0.047 and it was estimated from a subject with 90% of valid EEG signal. The highest significance threshold was found to be equal to 0.070 and it was found from a recording with 50% of valid EEG. A table showing the significance thresholds at *α* = 0.05 for a given area (Middle Central) for each subject, is reported in the Supplementary Material. The observed values were not homogeneously distributed across brain regions and different patterns were observed in the different tasks. In Figs 1A and 2A the median MIC values and the median MIC-*ρ*^2^ values across all regions and tasks are shown, respectively. Moreover, the statistical comparison between MIC and MIC-*ρ*^2^ values observed in the FB and BH was estimated for each region. Concerning MIC analysis, Fig. 1 show the topographic maps of average MIC values across all subjects in delta and alpha bands, respectively. The results of Wilcoxon sign rank test between the two tasks are also reported (see Fig. 1B). Specifically, in delta band the comparison between the two tasks was always significant (p < 0.05) in all regions except in the LP one where p was 0.067 and RC where p was 0.147. The MIC increased during the BH task, and, in particular, the higher increase was found in the middle-right anterior (MA and RA) regions and the middle-left central (MC and LC) regions. Conversely, in alpha band the comparison between the two tasks was never statistically significant. Concerning the analysis more specifically related to the nonlinear component, an average increase of MIC-*ρ*^2^ was found during the BH task, in both frequency bands. Differently from MIC, MIC-*ρ*^2^ showed more significant statistically differences in alpha band than in delta band. More in detail, the nonlinear correlation between EEG power and P_ET_CO_2_ significantly increased during the BH task in the MA area for both bandwidths and in the whole anterior brain area, as well as in the right central (RC) and in the middle posterior (MP) regions considering only the alpha band. Of note, in the Supplementary Information all MIC and MIC-*ρ*^2^ values and p-values can be found.

**Figure 3:**
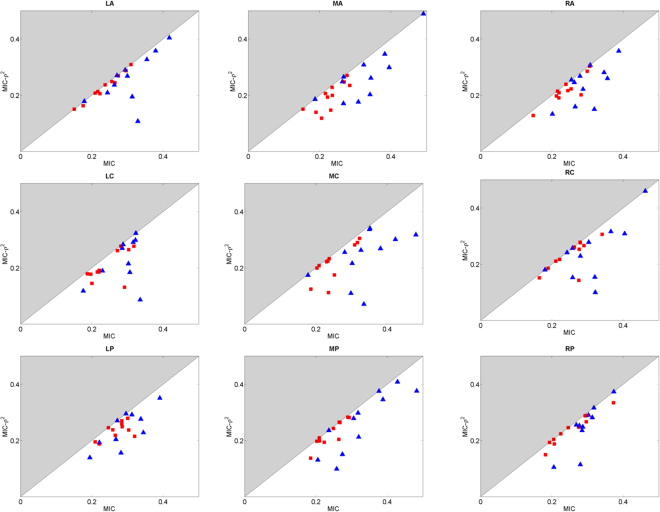
Scatter plots of MIC-*ρ*^2^ vs MIC as estimated in FB task (red squares) and in BH task (blue triangles) in the delta band for each scalp region.

**Figure 4:**
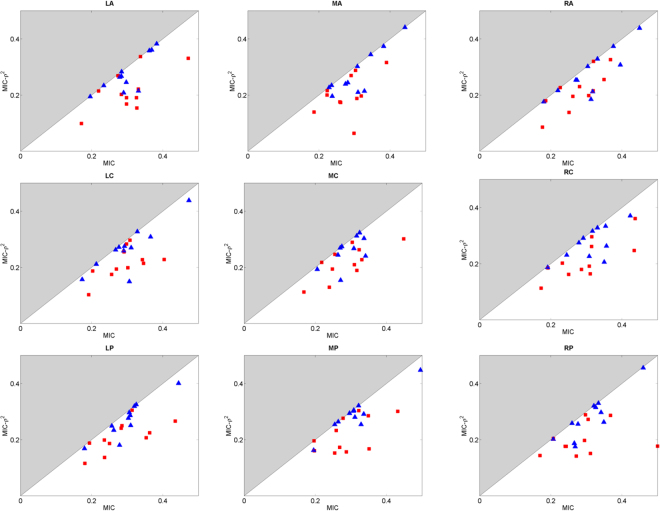
Scatter plots of MIC-*ρ*^2^ vs MIC as estimated in FB task (red squares) and in BH task (blue triangles) in the alpha band for each scalp region.

In Figs 3 and 4 the scatter plot of the reported MIC and MIC-*ρ*^2^ values are shown for delta and alpha band, respectively. Different values of sum of squared differences were observed. Specifically, larger deviations from the bisector were observed in the BH task with respect to the FB task in the delta band. In Table 1 the distances of the scatter points from the bisector are shown. A larger number of significant differences were found in the delta band (LA, RA, MP, MC) with respect to those estimated in the alpha band (MP).

**Table 1:** Distances of scatter points in the (MIC, MIC-*ρ*^2^) plane from the bisector: Wilcoxon test results (*p* values) for the difference between conditions (*α* = 0.05) and the value of the sign rank test statistic (*W*).

Freq. Band	LA	MA	RA	LC	MC	RC	LP	MP	RP
*δ*	*p* = 0.024	*p* = 0.365	*p* = 0.019	*p* = 0.206	*p* = 0.019	*p* = 0.067	*p* = 0.365	*p* = 0.024	*p* = 0.206
	*W* = 8	*W* = 22	*W* = 7	*W* = 18	*W* = 7	*W* = 12	*W* = 22	*W* = 8	*W* = 18
*α*	*p* = 0.083	*p* = 0.101	*p* = 0.320	*p* = 0.083	*p* = 0.054	*p* = 0.175	*p* = 0.054	*p* = 0.042	*p* = 0.067
	*W* = 53	*W* = 52	*W* = 45	*W* = 53	*W* = 55	*W* = 49	*W* = 55	*W* = 56	*W* = 54

## Discussion

In this study, the relationship between end tidal carbon dioxide (P_ET_CO_2_) and EEG power was explored in delta and alpha bands in nine different cortical regions of eleven healthy subjects during free breathing (FB) and voluntary breath hold (BH) tasks. Specifically, we studied the generic, i.e. linear and nonlinear, interaction by means of the Maximal Information Coefficient(MIC). We found that the wider oscillations in CO_2_ levels obtained during BH as compared to FB were associated with a change in the delta band power widely involving the anterior and central regions of the cortex, with both linear (predominant) and nonlinear relationships between P_ET_CO_2_ and EEG activity. On the other hand, during the same maneuver, the change in the alpha band was observed in a more restricted region (stronger in the middle anterior area) and was characterized by a mainly nonlinear relationship between P_ET_CO_2_ and EEG activity.

The main methodological novelty of the study has been the application of MIC (a generic linear and nonlinear correlation measure) and MIC-*ρ*^2^ (a “pure” nonlinear correlation measure) indexes^37^. This choice was motivated by the need of introducing a more general measure of the coupling between brain and respiratory activity. In fact, brain-respiratory interactions cannot be considered purely linear, since biological function in humans involves many nonlinear oscillations and feedbacks at different system levels (as cardiac, neural, respiratory, and endocrine)^41^. Furthermore, we analysed the regional effect of CO_2_ changes to highlight the dynamics of specific areas involved by bulbo-cortical pathways. In this work, the analysis of EEG activity was limited to the delta and alpha bands, since they seem to be highly influenced by hypercapnic stimuli^25,26,34,36,42,43^. The estimated MIC values were found to be significant in all areas and conditions. The significance of our results could have been inferred from the tables in^37^ where the significance level are computed for various MIC scores at a maximum size of 760 samples. Nonetheless, surrogate data analysis was performed using the approach suggested in^37^.

Considering the results obtained in the delta band, MIC indexes were found to be different between FB and BH tasks in all regions except the RC and LP. Specifically, significantly higher values were found in the BH condition with respect to FB, meaning an increase of generic coupling between P_ET_CO_2_ changes and EEG activity in this frequency band. The difference between the two conditions was higher in the middle and right anterior regions (MA and RA), as well as in the LC. By comparing MIC and MIC-*ρ*^2^ results, it is possible to hypothesize a major difference of linear coupling, between the FB and BH tasks: in fact, the increase was observed mainly using the MIC index while the “pure” nonlinear index MIC-*ρ*^2^, highlighted differences only in the MA region. These results were consistent with previous findings^43^, although it was possible this time to define a more precise distribution of the delta activity mainly localized in the central and anterior sections of the scalp and to identify also a nonlinear component limited to the MA region. As regards the alpha band, MIC was not different between FB and BH tasks. On the contrary, MIC-*ρ*^2^ was found to be statistically different between the two conditions in LA, MA, RA, RC and MP. Thus, in this band only changes in the non linear coupling seem to be identifiable between the two tasks, being the nonlinear coupling again higher in the BH with respect to FB condition. The scatter plot of MIC-*ρ*^2^ vs MIC was used trying to classify the two tasks. Indeed, in^43^, it was possible to highlight a more discriminative power of the MIC respect to the MIC-*ρ*^2^ to distinguish BH and FB in delta band. On the other hand, in the alpha band, MIC-*ρ*^2^ allowed to discriminate better the two tasks. Here, considering the scatter plot and the distance from the bisector of the Cartesian plane, differences in the delta band between FB and BH tasks were found to be significant in LA, RA, MC and MP, while in alpha band only the difference in MP was significant. Considering the value of W, generally, if *W* ≥ 56 means that the distance from the bisector is higher in FB and thus, in this condition, the linear component is more relevant. On the other hand, if *W* ≤ 10 the distance from bisector decreases and this means that in BH the linear component is more important. Looking at results in Table 1, W-values are less than 10 in delta band in several areas, confirming a more linear correlation in the BH task. Interestingly, in the MA region where both MIC and MIC-*ρ*^2^ indexes allow to discriminate both tasks, the distance from the bisector does not differ. This observation allow us to hypothesize that, in this region, the changes in the nonlinear relationship account for the difference between the two conditions. In the alpha band, W-values are higher, meaning that the linear correlation is stronger during FB. The latter observation together with MIC and MIC-*ρ*^2^ differences highlight a change in the quality of correlation between P_ET_CO_2_ and EEG activity in the alpha band from FB to BH tasks, passing from a simple one, i.e. linear, to a more complex interaction. The increase in the correlation between EEG activity and P_ET_CO_2_ during BH task may be related to chemoreflex^44^. In the FB tasks the small random variations of CO_2_ may produce only minor stimulation of the chemoreflex, with small perturbation of the neural system. During BH, the higher changes in CO_2_ levels lead to increased stimulation of the chemoreflex. This may in turn increase the firing not only to the pre-Bötzinger complex in the medulla, but also to upper centres via bulbo-(thalamic)-cortical pathways. Indeed, the involvement of the anterior thalamus and ventral posterior lateral and ventrolateral thalamic nuclei, as well as the posterior putamen has been demonstrated in the response to CO_2_ variations. Furthermore, different thalamocortical connections related to the respiratory control have been described^9^. These ascending pathways have been related to the neural perception of dyspnea, but may also represent an increased neural output to the respiratory and autonomic systems mediated by the cortex^45^. The same pathways may be responsible of arousal response during apneas, being thalamus and cortex the final common pathways for arousal. In humans, the delta band is usually associated with autonomic and metabolic process suggesting an involvement in the integration of cerebral activity with homeostatic processes. This kind of oscillation is relevant in the early developmental stages and during slow-wave sleep. A hypothesis is that delta activity represents an evolutionary old oscillatory mode present in lower vertebrates and replaced by more advanced process with higher frequency oscillation in walking adult humans^46^. During hypercapnia related to BH, the generic linear increasing in MIC index with respect to FB with the increasing in P_ET_CO_2_ could represent an index of the suppression of a more complex activity (higher oscillatory brain wave). Thus, this dynamics could reflect a condition in which the brain is shifted from high frequencies (alpha, beta and gamma spectra), associated with complex neuronal tasks, to low frequencies associated with control of vital function, such as control of respiration and hemodynamics. The MIC analysis employed in this study, differently from cross-correlation analysis, is dimensionless and due to calculation complexities it was limited to the comparison between the whole FB task and the whole BH task. Therefore, it is not possible to establish whether the increase of CO_2_ during apneas would be associated with an increase rather with a decrease in the delta or alpha band. Furthermore, we did not explore the temporal relationship between the two signals. However, this analysis might complement a linear one, such that based on the study of the cross correlation coefficient, that can partially explain the whole complexity of the system. In conclusion, this study investigated the generic, both linear and non-linear, mechanisms underlying the neuronal response to hypercapnia using MIC and MIC-*ρ*^2^ indexes. Looking at the results, in delta band a generic behaviour can be observed, while a prominent nonlinear coupling seems to be present in alpha band during BH task respect to FB. These results can provide interesting clues toward the comprehension of the relationship between cortical activity and CO_2_ variations and they highlight the importance of taking into account also nonlinear brain-respiratory coupling.

## Methods

### Experimental protocol

Eleven healthy subjects (all males, age 30 ± 6) underwent the experimental protocol. EEG signal and physiological data (such as exhaled CO_2_ and Oxygen saturation (SpO_2_)) were simultaneously recorded. Subjects had to lie down with eyes closed staying awake. Two tasks were performed, each for a duration of 6 minutes. In the first one, the subjects had to breath normally while all signals were recorded. We referred to this task as free breathing (FB) task. In the second one, after a minute of FB, subjects had to alternate 30 seconds of voluntary breath hold and 30 seconds of FB (five times to reach the 6 minutes period). We referred to this task as breath hold (BH) task. All the acquisitions were conducted under controlled conditions and the subjects were previously trained to perform the 30 seconds BH. Indeed, they had to hold their breath feeling any discomfort or excessive air hunger avoiding great movements. For this reason, inspiratory BH is preferable as compared to expiratory BH. To start the BH period, an operator gave a signal to the subject simply touching his leg. To terminate the task, the operator touched again the leg’s subject. The experimental protocol was approved by the Ethical Committee of the University of Pisa-Pisa University Hospital, Pisa (Italy). The recordings were carried out in agreement with the Declaration of Helsinki. Written informed consent was obtained from all subjects.

### EEG signal and physiological data acquisition

A 64 EEG device (Compumedics Neuroscan, SynAmps RT) was used. The electrodes were mounted into an elastic cap according to the standard 10–20 system. The EEG device also included two electrodes for ocular movement detection and two for the registration of the electrocardiographic signal. A reference channel was located between CZ and PZ, while the ground channel was situated between FPZ and FZ. The impedance of each electrode was maintained below 30 kΩ during the all acquisitions. The signal was acquired using low-pass filtered at 400 Hz and a sampling rate of 1 kHz. Exhaled CO_2_ was recorded using a CO_2_ analyzer (Cosmoplus; Novametrics) and SpO_2_ was recorded with a pulse oximeter (Pulsox-7; Minolta). All the data were digitized through a National Instrument acquisition card and an home-made software written in Java and developed in our institution.

### EEG processing

The recorded EEG signals were analysed using the proprietary software Curry Neuroimaging Suite 7. All channels were re-referenced to the average signal. A baseline correction was applied to remove a constant or linear DC offset from the data. All signals were filtered using a band pass filter between 1 and 30 Hz. The channels with low quality signal were excluded from the analysis. Blink and cardiac artefacts were detected with threshold method and reduced using a Principal Component Analysis (PCA) method^47^. A visual inspection of the processed signals was performed. The signal intervals showing not reducible movement artefacts were detected and excluded from the analysis. A team of Neurologists assessed the quality of the remaining EEG signal for the further analysis. In MATLAB (MathWorks, Natick MA), the spectrogram of each channel was computed applying an Hanning window of 2 seconds with overlapping of 1 second. Nine different areas were extracted dividing the cap into three different sections (left L, middle M and right R) and further dividing into three rostrocaudal regions (anterior A, central C and posterior P). The nine areas were named as left anterior (LA), middle anterior (MA), right anterior (RA), left central (LC), middle central (MC), right central (RC), left posterior (LP), middle posterior (MP) and right posterior (RP). The local field power of each area was found averaging the power of all channels in the area of interest. Two EEG power bands were analysed, such as delta (1–4 Hz) and alpha (8–13 Hz) bands. It was demonstrated that these EEG frequency bands are sensitive to hypercapnic stimulation^25,26^. Finally, EEG power signals were linearly detrended and resampled at 50 Hz.

### Physiological Parameters

Starting from the recorded CO_2_ signal, P_ET_CO_2_ was extracted as maximum value of CO_2_ at the end of each expiration. A cubic spline model was use to interpolate the CO_2_ values and in particular, to estimate the P_ET_CO_2_ also during BH period. To optimize the synchronization between P_ET_CO_2_ and EEG signals, as for EEG power, P_ET_CO_2_ was linearly detrended and resampled at 50 Hz. SpO_2_ was recorded and use to verify whether the task produce some effects on oxygen blood level.

### Maximal Information Coefficient (MIC)

To consider only the contribution of the brain-respiratory slowly components, EEG power signals and respiratory signals were smoothed using a zero-phase moving averaged filter of 10 s^48^. To quantify the generic, i.e. linear and nonlinear, relationship between EEG power and P_ET_CO_2_, Maximal Information Coefficient (MIC) was calculated^37^. MIC meets two heuristic properties (as generality and equitability) that allow to observe both functional and no functional relationship with a score that roughly equals the coefficient of determination (*R*^2^) of the data relative to the regression function^37,38,49^. Generally, considering two variable data *x* and *y*, if a relationship between each other exist, a grid on the scatter plot of the two variables can be created. First of all, the MIC algorithm finds the *x*−*y* grid (G) with the highest induced mutual information. For each resolution, the best grid and the normalized score are stored to compile the characteristic matrix *M* = (*m*_*x*,*y*_) where every *m*_*x*,*y*_ is the highest normalized mutual information of any *x*, *y* grid. Visualizing this matrix as surface, MIC represents the highest point. Formally, for a grid G, *I*_*G*_ is the mutual information of the probability distribution on that particular grid G and *m*_*x*,*y*_ can be derived as

1 $${m}_{x,y}=\frac{max\{{I}_{G}\}}{log\,min\{{m}_{x,y}\}}$$

Considering that the sample size is n and B is a function of the sample size (B = n^0.6^), MIC can be defined as:

2 $$MIC=\mathop{{\rm{\max }}}\limits_{xy < B}({m}_{x,y})$$

To test the statistical significance of the MIC, we performed surrogate data analysis as described in^37^. Briefly, surrogate data were created choosing 500 random permutations of EEG signal and P_ET_CO_2_. The critical values of MIC, corresponding to *α* = 0.05, were estimated from the 95th percentile of the values obtained from random permutations. To evaluate the weight of the nonlinear component of the interaction between the two variables, MIC-*ρ*^2^ was estimated, where *ρ*^2^ is the Pearson coefficient. This index is always smaller that MIC, and it was proposed for the detection of nonlinear relationships, being less sensitive to linear interactions than the MIC coefficients^37^. We have to stress that even in the case of a MIC-*ρ*^2^ close to zero, a linear coupling between variables cannot be excluded. For these reasons, the joint analysis of MIC-*ρ*^2^ and MIC indexes, is useful to investigate the nature of the interaction. For instance, if a high value of MIC and a concomitant low value of MIC-*ρ*^2^ are observed, the interaction is likely to be mainly linear. On the other hand, if high values of MIC and MIC-*ρ*^2^ are observed the weight of the linear component is lower with respect to the non linear one.

### Statistical Analysis

The comparison between FB and BH tasks were performed both for MIC and MIC-*ρ*^2^. Specifically, statistical significance was evaluated using non-parametric Wilcoxon signed rank test (W)^50^. The null hypothesis is that the differences between FB and BH tasks both in MIC as well as MIC-*ρ*^2^ come from a distribution with zero median. Scatter plots of MIC-*ρ*^2^ vs MIC, observed for each subject, will be drawn to explore the relevance of linear and nonlinear interactions. Since, MIC is always larger than MIC-*ρ*^2^, the scatter plot will lie below the bisector. If for a given subject, the pure non-linear component will differ from the generic (linear and non linear) relationship, the corresponding point in the scatter plot will move from the bisector. As a measure of the difference between the two indexes across the different subjects, the distance between the points of scatter plot and the bisector of the Cartesian plane (i.e. corresponding to equal values of MIC and MIC-*ρ*^2^) will be evaluated for subject, condition and brain area. The statistical difference of this measure between the FB and BH tasks, will be evaluated with a Wilcoxon signed rank test.

### Data availability

The raw EEG data of this study are not publicly available due to ethical restrictions, however they can be reasonable requested from the corresponding author. All data generated from the raw EEG data (i.e., correlation results) are included in the Supplementary Information files.

## Electronic supplementary material

Supplementary material
