# Supplementary material for: Analysis of generic coupling between EEG activity and PETCO2 in free breathing and breath-hold tasks using Maximal Information Coefficient (MIC)
Source: Sci Rep. 2018 Mar 14;8:4492. doi: 10.1038/s41598-018-22573-6 (PMC5851981; doi:10.1038/s41598-018-22573-6)
Supplement: Supplementary file 1 — Supplementary material [file 41598_2018_22573_MOESM1_ESM.pdf]

Analysis of generic coupling between EEG activity and $P_{ET}CO_2$ in free breathing and breath-hold tasks using Maximal Information Coefficient (MIC)

Maria Sole Morelli[1,2,*], Alberto Greco[2,3], Gaetano Valenza[2,3], Alberto Giannoni[4], Michele Emdin[1,4], Enzo Pasquale Scilingo[2,3], and Nicola Vanello[2,3]

[1]Scuola Superiore Sant'Anna, Department of Life science, Pisa, 56127, Italy
[2]Research Center "E. Piaggio", University of Pisa, Pisa, 56122, Italy
[3]University of Pisa, Dipeartimento di Ingegneria dell'Informazione, 56124 Pisa,Italy
[4]Fondazione Toscana Gabriele Monasterio, National Research Council, 56124, Pisa, Italy
[*]mar.morelli@sssup.it

SUPPLEMENTARY INFORMATION

	MIC																	
	LA		MA		RA		LC		MC		RC		LP		MP		RP	
	FB	BH	FB	BH	FB	BH	FB	BH	FB	BH	FB	BH	FB	BH	FB	BH	FB	BH
1	0.22	0.31	0.15	0.34	0.22	0.29	0.19	0.31	0.21	0.33	0.19	0.33	0.25	0.27	0.21	0.27	0.19	0.28
2	0.15	0.30	0.21	0.34	0.15	0.28	0.20	0.32	0.19	0.30	0.16	0.40	0.21	0.31	0.19	0.43	0.21	0.30
3	0.26	0.18	0.22	0.19	0.22	0.20	0.22	0.18	0.25	0.18	0.28	0.18	0.28	0.19	0.26	0.21	0.30	0.21
4	0.24	0.33	0.24	0.31	0.25	0.32	0.29	0.34	0.24	0.30	0.28	0.32	0.27	0.28	0.22	0.26	0.23	0.27
5	0.22	0.29	0.22	0.26	0.24	0.31	0.28	0.29	0.23	0.35	0.28	0.26	0.30	0.39	0.29	0.32	0.29	0.28
6	0.26	0.35	0.23	0.38	0.28	0.36	0.22	0.32	0.32	0.33	0.29	0.37	0.30	0.34	0.25	0.39	0.20	0.32
7	0.18	0.24	0.19	0.27	0.21	0.27	0.22	0.23	0.24	0.42	0.21	0.26	0.22	0.22	0.20	0.32	0.25	0.31
8	0.31	0.27	0.28	0.32	0.30	0.26	0.27	0.32	0.32	0.28	0.34	0.24	0.28	0.27	0.27	0.24	0.27	0.29
9	0.30	0.42	0.27	0.49	0.31	0.39	0.32	0.53	0.31	0.48	0.28	0.46	0.26	0.62	0.29	0.48	0.30	0.50
10	0.27	0.38	0.28	0.39	0.24	0.35	0.30	0.30	0.23	0.38	0.26	0.30	0.32	0.34	0.27	0.38	0.37	0.37
11	0.21	0.26	0.23	0.27	0.22	0.25	0.20	0.29	0.20	0.35	0.22	0.28	0.28	0.30	0.21	0.31	0.18	0.28
p	0.014		0.002		0.007		0.010		0.014		0.147		0.067		0.014		0.042	
W	6		1		4		5		6		16		12		6		10	

Table 1. MIC values recorded during FB and BH task in the nine regions in Delta Band. In last two rows the p-value of a two-sided (p) Wilcoxon signed rank test ($\alpha = 0.05$)and the value of the sign rank test statistic (W) are reported.

MIC																		
	LA		MA		RA		LC		MC		RC		LP		MP		RP	
	FB	BH	FB	BH	FB	BH	FB	BH	FB	BH	FB	BH	FB	BH	FB	BH	FB	BH
1	0.33	0.23	0.30	0.24	0.31	0.22	0.35	0.21	0.33	0.27	0.29	0.29	0.35	0.26	0.35	0.26	0.37	0.26
2	0.17	0.28	0.19	0.31	0.18	0.30	0.19	0.28	0.17	0.32	0.17	0.32	0.18	0.31	0.20	0.31	0.17	0.33
3	0.30	0.20	0.22	0.23	0.23	0.18	0.30	0.17	0.25	0.21	0.23	0.19	0.25	0.18	0.26	0.20	0.24	0.21
4	0.27	0.33	0.30	0.33	0.28	0.40	0.34	0.29	0.30	0.27	0.31	0.31	0.28	0.28	0.29	0.31	0.30	0.27
5	0.33	0.28	0.26	0.24	0.25	0.27	0.26	0.29	0.25	0.26	0.31	0.24	0.24	0.26	0.27	0.25	0.27	0.33
6	0.30	0.37	0.32	0.35	0.32	0.33	0.29	0.33	0.31	0.32	0.31	0.33	0.29	0.33	0.32	0.32	0.29	0.32
7	0.28	0.29	0.26	0.31	0.26	0.31	0.27	0.31	0.24	0.34	0.25	0.36	0.24	0.31	0.25	0.33	0.24	0.34
8	0.33	0.28	0.31	0.27	0.35	0.27	0.30	0.27	0.32	0.31	0.43	0.28	0.43	0.30	0.35	0.31	0.31	0.27
9	0.47	0.36	0.39	0.44	0.37	0.45	0.40	0.47	0.45	0.51	0.44	0.42	0.36	0.44	0.43	0.49	0.50	0.46
10	0.34	0.38	0.29	0.38	0.32	0.38	0.31	0.37	0.32	0.34	0.31	0.35	0.31	0.31	0.28	0.34	0.30	0.35
11	0.22	0.30	0.22	0.28	0.19	0.32	0.20	0.31	0.22	0.27	0.19	0.35	0.19	0.32	0.20	0.30	0.21	0.28
p	1.000		0.147		0.175		0.700		0.320		0.700		0.638		0.365		0.465	
W	33		16		17		28		21		28		27		22		24	

Table 2. MIC values recorded during FB and BH task in the nine regions in Alpha Band. In last two rows the p-value of a two-sided (p) Wilcoxon signed rank test ($\alpha = 0.05$)and the value of the sign rank test statistic (W) are reported.

MIC-ρ^2																		
	LA		MA		RA		LC		MC		RC		LP		MP		RP	
	FB	BH	FB	BH	FB	BH	FB	BH	FB	BH	FB	BH	FB	BH	FB	BH	FB	BH
1	0.21	0.19	0.15	0.20	0.21	0.22	0.18	0.18	0.21	0.07	0.19	0.15	0.25	0.20	0.20	0.15	0.19	0.11
2	0.15	0.27	0.12	0.26	0.13	0.27	0.15	0.29	0.12	0.22	0.15	0.31	0.19	0.29	0.14	0.41	0.19	0.29
3	0.24	0.18	0.19	0.19	0.19	0.13	0.19	0.12	0.17	0.17	0.25	0.18	0.25	0.14	0.20	0.13	0.29	0.11
4	0.24	0.11	0.20	0.18	0.22	0.15	0.13	0.09	0.11	0.11	0.14	0.10	0.22	0.16	0.19	0.10	0.22	0.26
5	0.21	0.29	0.21	0.25	0.24	0.31	0.28	0.27	0.22	0.34	0.28	0.26	0.24	0.35	0.28	0.30	0.29	0.25
6	0.25	0.33	0.23	0.35	0.20	0.26	0.19	0.30	0.29	0.26	0.27	0.32	0.28	0.23	0.24	0.35	0.20	0.32
7	0.16	0.21	0.14	0.17	0.20	0.16	0.19	0.19	0.23	0.30	0.21	0.15	0.19	0.19	0.20	0.21	0.25	0.28
8	0.31	0.27	0.23	0.31	0.29	0.25	0.26	0.32	0.30	0.26	0.31	0.24	0.27	0.27	0.26	0.24	0.25	0.25
9	0.29	0.40	0.25	0.49	0.30	0.36	0.28	0.44	0.28	0.32	0.28	0.46	0.24	0.40	0.28	0.38	0.27	0.50
10	0.27	0.36	0.27	0.30	0.22	0.28	0.26	0.22	0.22	0.27	0.26	0.28	0.22	0.28	0.27	0.38	0.33	0.37
11	0.21	0.24	0.15	0.27	0.21	0.25	0.18	0.28	0.20	0.34	0.22	0.23	0.26	0.30	0.21	0.28	0.15	0.24
p	0.278		0.005		0.320		0.278		0.365		0.831		0.520		0.320		0.240	
W	20		3		21		20		22		36		25		21		19	

Table 3. MIC-ρ^2 values recorded during FB and BH task in the nine regions in Delta Band. In last two rows the p-value of a two-sided (p) Wilcoxon signed rank test ($\alpha = 0.05$)and the value of the sign rank test statistic (W) are reported.

	MIC-ρ^2																	
	LA		MA		RA		LC		MC		RC		LP		MP		RP	
	FB	BH	FB	BH	FB	BH	FB	BH	FB	BH	FB	BH	FB	BH	FB	BH	FB	BH
1	0.15	0.23	0.06	0.23	0.20	0.22	0.21	0.21	0.23	0.27	0.18	0.29	0.21	0.23	0.29	0.26	0.29	0.26
2	0.10	0.28	0.14	0.30	0.09	0.30	0.10	0.27	0.11	0.31	0.11	0.32	0.12	0.30	0.16	0.28	0.14	0.33
3	0.17	0.19	0.20	0.23	0.23	0.18	0.28	0.16	0.25	0.19	0.20	0.19	0.19	0.17	0.23	0.16	0.18	0.20
4	0.27	0.21	0.29	0.21	0.23	0.31	0.23	0.28	0.29	0.15	0.26	0.23	0.24	0.18	0.16	0.30	0.29	0.19
5	0.19	0.27	0.18	0.20	0.14	0.25	0.17	0.26	0.19	0.24	0.19	0.23	0.14	0.25	0.17	0.25	0.14	0.32
6	0.19	0.36	0.20	0.34	0.21	0.33	0.26	0.33	0.21	0.32	0.16	0.33	0.25	0.32	0.30	0.32	0.20	0.32
7	0.20	0.21	0.17	0.21	0.20	0.18	0.19	0.15	0.13	0.24	0.16	0.26	0.20	0.25	0.15	0.25	0.18	0.30
8	0.22	0.26	0.19	0.24	0.26	0.25	0.20	0.26	0.19	0.27	0.25	0.27	0.27	0.28	0.17	0.31	0.15	0.18
9	0.33	0.36	0.32	0.44	0.33	0.44	0.23	0.44	0.30	0.48	0.36	0.37	0.22	0.40	0.30	0.45	0.18	0.46
10	0.34	0.38	0.27	0.37	0.32	0.37	0.30	0.31	0.26	0.30	0.30	0.33	0.30	0.29	0.28	0.29	0.27	0.26
11	0.21	0.25	0.22	0.24	0.18	0.21	0.19	0.27	0.22	0.27	0.19	0.21	0.19	0.32	0.20	0.29	0.20	0.26
p	0.019		0.014		0.024		0.083		0.083		0.019		0.054		0.019		0.054	
W	7		6		8		13		13		7		11		7		11	

Table 4. MIC-ρ^2 values recorded during FB and BH task in the nine regions in Alpha Band. In last two rows the p-value of a two-sided (p) Wilcoxon signed rank test ($\alpha = 0.05$)and the value of the sign rank test statistic (W) are reported.

Table 5. The significance thresholds of MIC at $\alpha = 0.05$ for a given area (Middle Central) for each subject. The critical values were estimated from 95th percentile of the value obtained from random permutations.

	Free breathing		Breath Hold	
Sub	Delta Band	Alpha Band	Delta Band	Alpha Band
1	0,0536	0,0537	0,0589	0,0588
2	0,0537	0,0538	0,0620	0,0620
3	0,0583	0,0585	0,0480	0,0479
4	0,0658	0,0658	0,0532	0,0529
5	0,0602	0,0603	0,0610	0,0621
6	0,0612	0,0613	0,0629	0,0631
7	0,0542	0,0542	0,0556	0,0560
8	0,0613	0,0618	0,0563	0,0561
9	0,0563	0,0569	0,0695	0,0697
10	0,0597	0,0595	0,0651	0,0661
11	0,0555	0,0555	0,0559	0,0564
